# Characterization of a novel β-agarase from Antarctic macroalgae-associated bacteria metagenomic library and anti-inflammatory activity of the enzymatic hydrolysates

**DOI:** 10.3389/fmicb.2022.972272

**Published:** 2022-09-02

**Authors:** Xiaoqian Gu, Luying Zhao, Jiaojiao Tan, Qian Zhang, Liping Fu, Jiang Li

**Affiliations:** ^1^Key Laboratory of Ecological Environment Science and Technology, First Institute of Oceanography, Ministry of Natural Resources, Qingdao, China; ^2^CAS and Shandong Province Key Laboratory of Experimental Marine Biology, Center for Ocean Mega-Science, Institute of Oceanology, Chinese Academy of Sciences, Qingdao, China

**Keywords:** metagenomic, agarase, biochemical characterization, agaro-oligosaccharides, anti-inflammatory

## Abstract

An agarase gene (*aga1904*) that codes a protein with 640 amino acids was obtained from the metagenomic library of macroalgae-associated bacteria collected from King George Island, Antarctica. Gene *aga1904* was expressed in *Escherichia coli* BL21 (DE3) and recombinant Aga1904 was purified by His Bind Purification kit. The optimal temperature and pH for the activity of Aga1904 were 50°C and 6.0, respectively. Fe^3+^ and Cu^2+^ significantly inhibited the activity of Aga1904. The *V*_max_ and *K*_*m*_ values of recombinant Aga1904 were 108.70 mg/ml min and 6.51 mg/ml, respectively. The degradation products of Aga1904 against agarose substrate were mainly neoagarobiose, neoagarotetraose, and neoagarohexaose analyzed by thin layer chromatography. The cellular immunoassay of enzymatic hydrolysates was subsequently carried out, and the results showed that agaro-oligosaccharides dominated by neoagarobiose significantly inhibited key pro-inflammatory markers including, nitric oxide (NO), interleukins 6 (IL-6), and tumor necrosis factor α (TNF-α). This work provides a promising candidate for development recombinant industrial enzyme to prepare agaro-oligosaccharides, and paved up a new path for the exploitation of natural anti-inflammatory agent in the future.

## Introduction

Agar is a natural polysaccharide derived from the cell walls of red algae (Marinho-Soriano, 2001), primarily *Gracilaria* and *Gelidiaceae* (Lemus et al., 1991). Agar has found wide application in food, biomedical, and chemical industries owing to its jelly-like character at ambient temperature, excellent rheological properties, good compatibility with other polysaccharides, and low cost (Ramawat and Merillon, 2015). Agar is also commonly used as a gelling agent in culture media for bacteriological studies (Cregut and Rondags, 2013). However, its high viscosity, low water solubility, and low bioavailability limit its application (Zhu et al., 2021).

Marine oligosaccharides have drawn increasing attention owing to their biological activity, good solubility, and excellent bioavailability (Zhu et al., 2021). Studies have shown that agaro-oligosaccharides, the degradation products of agar polysaccharides, have many potential applications. Agaro-oligosaccharides can inhibit the activity of α-glucosidase and thus inhibit the rise of blood glucose (Chen et al., 2021); serve as a new prebiotic; promote growth of beneficial intestinal bacteria, such as *Bifidobacterium* and *Lactobacillus*, thereby inhibiting the growth of pathogenic bacteria (Hu et al., 2006); serve as an antitumor agent by blocking the cell cycle in the S phase, which inhibits angiogenesis by promoting umbilical vein endothelial cell apoptosis (Enoki et al., 2012); inhibit the production of reactive oxygen species in liver cells, thereby reducing oxidative cell damage (Chen et al., 2006); and block the expression of inducible nitric oxide synthase (iNOS), which inhibits excess nitric oxide (NO) production and reduces oxidative damage, and can therefore be used to treat inflammatory diseases, such as arthritis (Takara Co, 1998). Furthermore, Enoki et al. (2002) have developed a food preservative composed of agaro-oligosaccharides, which can effectively prevent discoloration, corruption and oxidation. In recent years, research on acidolysis to obtain agaro-oligosaccharides has enabled production of commercial products of agaro-oligosaccharides in Japan. TaKaRa Agaoligo™ (TaKaRa Bio, Shiga, Japan), an agaro-oligosaccharide product obtained from acidolysis of agar, is composed of neoagarobiose, neoagarotetraose and neoagarohexaose. It has antioxidant effects, which may prevent some diseases related to oxidation, such as chronic renal dysfunction, ulcerative colitis, arthritis and rheumatoid arthritis, cataract, glaucoma, and tumors caused by gene damage (Weinberger et al., 2001).

Several studies have suggested that the anti-inflammatory activity of agaro-oligosaccharides is related to the degree of structural polymerization. Agaro-oligosaccharides consist of neoagarooligosaccharides (NAOSs) and agarooligosaccharides (AOSs), which have α-1,3-linked-3,6-anhydro-L-galactose (L-AHG) and β-1,4-linked-D-galactose (D-Gal) as their nonreducing ends, respectively (Jiang et al., 2021). Yun et al. (2013) tested the anti-inflammatory activity of several agaro-oligosaccharides, including neoagarobiose; D-galactose, D-Gal3,6-anhydro-L-galactose (L-AHG); and D-galactose, D-Gal3,6-anhydro-D-galactose (D-AHG). Only L-AHG showed significant anti-inflammatory activity, which suggests that L-AHG at the nonreducing end of agaro-oligosaccharides is required for anti-inflammatory activity. Wang et al. (2017) found that the neoagaro-series, especially NAOSs, can reduce the production and release of NO in lipopolysaccharide (LPS)-induced mouse macrophages by inhibiting the expression and secretion of iNOS. Furthermore, they reduced the production of pro-inflammatory cytokines, therefore showing significant anti-inflammatory effects (Wang et al., 2017). Zou et al. (2019) evaluated the effect of different degrees of agaro-oligosaccharide polymerization on anti-inflammatory activity *in vitro* using LPS-induced mouse-macrophage and zebrafish models. The results showed that unpurified oligosaccharides exhibit higher anti-inflammatory activity owing to their synergistic effects. This could simplify the preparation, separation, and purification of agaro-oligosaccharides, which enhances their application potential in the food, medicine, and cosmetic industries (Zou et al., 2019). Although studies regarding the anti-inflammatory activity of agaro-oligosaccharides have previously been performed, the correlation between anti-inflammatory activity and polymerization remains unclear.

Agaro-oligosaccharides can be prepared through enzymatic, physical, and chemical degradation of agar. Although acidolysis has been widely used in the preparation of agaro-oligosaccharides, the disadvantage of this method is that the end products are complicated and difficult to control (Li et al., 2007). In addition, hydrochloric acid, sulfuric acid and other substances used in the degradation reaction are ecological hazards and pose a safety risk under high temperature conditions (Kazłowski et al., 2015; Xu et al., 2018). Compared with traditional chemical degradation, enzymatic hydrolysis has the advantage of substrate specificity, product specificity, mild reaction conditions, high yield, and high stability (Yang et al., 2014). Agarase is an enzyme that can degrade agarose into oligosaccharides and can be classified according to the type of glycosidic bond that it cleaves (Ekborg et al., 2006). α-Agarase cleaves the α-1,3-glycosidic bond of agarose to produce AOSs, whereas β-agarase cleaves the β-1,4-glycosidic bond to produce NAOSs (Ekborg et al., 2006). Currently, β-agarase accounts for the majority of reported agarases. The homology of coding genes indicates that β-agarases are mainly distributed in five different carbohydrate-active enzyme (CAZymes) families, including GH16, GH50, GH86, GH117, and GH118, whereas α-agarases mainly belong to the GH96 family. Although many agarases have been reported, few of them can be used for industrial application. It is therefore necessary to screen for enzymes with higher thermostability and catalytic activity.

Currently, novel enzymes are mainly obtained by screening activity of proteins isolated from culturable microbes. However, 99% of the microorganisms in nature cannot be cultured (Tian et al., 2012). Metagenomic technologies have provided a new way to screen novel enzymes, especially those produced by uncultured microbes (Bhattacharyya et al., 2014).

Macroalgae surfaces harbor large numbers of microbes (Cundell et al., 1977), which have been shown to synthesize many compounds that possess a wide variety of biological activities, including antitumor, antiviral, antibiotic, and dehalogenase activity (Martin et al., 2014). These macroalgae-associated microbes have also been shown to produce algae-specific polysaccharide-degrading enzymes, including agarases, carrageenases, and alginate lyases are therefore an important resource for the discovery of novel polysaccharide-degrading enzymes (Michel et al., 2013). Antarctica has extreme environmental conditions, such as low temperatures, high salinity, and strong radiation, and therefore rich marine biological resources and many unique macroalgae species. Among the 117 species of macroalgae reported from Antarctica, 57 are endemic to the Antarctic region (Ramírez, 2010). Therefore, macroalgae-associated microbes collected from Antarctica are expected to be a source of novel agarases.

In this study, an agarase gene (*aga1904*) was screened and cloned from the macroalgae-associated bacteria metagenomic library and expressed in *E. coli*. The purified recombinant agarase (Aga1904) was characterized, and agaro-oligosaccharides were prepared *via* enzymatic scission. We also investigated the effects of the agaro-oligosaccharides produced by Aga1904 on the production of inflammatory factors, including NO, interleukins 6 (IL-6), and tumor necrosis factor α (TNF-α), by LPS-stimulated RAW264.7 macrophages.

## Materials and methods

### Materials and reagents

Plasmid pET-30a (+) and *E. coli* BL21 (DE3) were purchased from Tiangen (Beijing, China). Agarose was purchased from Sinopharm Group Chemical Reagent (Shanghai, China). His-tag-specific purification kits were purchased from GE Healthcare (Pittsburgh, PA, United States). Thin layer chromatography (TLC) silica gel plates were purchased from Kadel (Shanghai, China). Bio-Gel P-2 was purchased from Bio-Rad (Pittsburgh, PA, United States). RAW 264.7 macrophages were obtained from the Cell Bank, Chinese Academy of Sciences (Shanghai, China). LPS and L-monomethylarginine (L-NMMA) were purchased from Yuanye (Shanghai, China). 3-(4,5-dimethylthiazol-2-yl)-2,5-diphenyltetrazolium bromide (MTT) and dimethyl sulfoxide (DMSO) were purchased from Sigma-Aldrich (St. Louis, MO, United States). Fetal bovine serum (FBS) was obtained from Gibco (Grand Island, NY, United States). Enzyme Linked Immunoabsorbent Assay (ELISA) kits (TNF-α and IL-6) were purchased from AndHider (Qingdao, Shandong, China).

### Construction of the metagenomic library

Macroalgae samples were collected from the Great Wall Station in Antarctica (Shengwu Cove, 58°W 62°S) during China’s 34th Antarctic expedition (Gui et al., 2020). Samples of six species of macroalgae, including *Melanthalia abscissa, Callithamnion tetragonum, Plocamium cartilagineum, Phaeurus antarcticus, Pachymenia orbicularis*, and *Desmarestia antarctica*, were removed from the refrigerator and placed in a glass dish. The algae surface was washed with sterilized seawater, cut into pieces and put it into sterile tubes. Sterile seawater was added and eddy oscillations were used to shock it three times for 2 min each. Microorganisms were then extracted and collected using 0.22-μm sterile filter membranes (50 mm). The genomic DNA from the collected bacteria samples was extracted use FastDNA Spin Kit for Soil (MP Bio, United States). To conduct metagenomic sequencing of the DNA samples of the macroalgae-associated microorganisms, the qualified genomic DNA was sent to Jingneng Biotechnology Co., Ltd. (Shanghai, China) for sequencing, assembly and functional annotation.

### Gene sequence analysis of *aga1904*

The *aga1904* gene sequence was obtained by screening the metagenomic library and used as the template to synthesize the target gene. Basic Alignment Search Tool (BLAST)^1^ was used to identify the *aga1904* sequence and the DNAMAN software package^2^ was used for multiple sequence alignment. The phylogenetic tree was constructed and analyzed with MEGA 5.0 software.

### Expression and purification

The presumptive agarase gene, *aga1904*, was optimized according to codon usage preference of prokaryotic host *E. coli* BL21. Then, the gene sequence without signal peptide and stop codon was synthesized by Nanjing Kingsley Biotechnology (Nanjing, China). The synthesis gene with *BamH* I and *Xba* I restriction endonuclease sites was ligated into vector pET30(a), which was previously digested with the same two restriction enzymes. The transformants exhibiting agarolytic activity were selected by detecting the formation of clear halos around colonies on LB plates after dyeing with the fresh Lugo iodine solution (5 g iodine, 20 g potassium iodide, and 100 ml distilled water).

*Escherichia coli* BL21 (DE3), containing the recombinant plasmid pET-30(a) + *aga1904*, was cultured in Luria-Bertani (LB) medium (containing 50 μg/ml kanamycin) at 37°C for 2–3 h. Once a cell concentration of 0.6–0.8 at OD_600_ had been obtained, 0.5 mM isopropyl-β-D-thiogalactopyranoside (IPTG) was added to induce fusion protein expression. The collected fermentation precipitate was suspended in phosphate buffer (20 mM, pH 7.0) and sonicated at 4°C for 30 min. The supernatant of the cell homogenate was harvested at 10 000 × *g* at 4°C for 20 min and subsequently loaded on a Ni-NTA His-tag Kit to purify the recombinant Aga1904. Binding buffer (10 mM imidazole, 50 mM NaH_2_PO_4_, 300 mM NaCl, pH 8.0) was used to wash the recombinant Aga1904. Elution buffer with different concentrations of imidazole (20, 80, 140, and 200 mM) was used to elute the recombinant Aga1904. The purified Aga1904 was analyzed with 12% sodium dodecyl sulfate polyacrylamide gel electrophoresis (SDS-PAGE).

### Agarase activity assay

The enzyme-substrate mixture, comprising 1 ml Aga1904 and 1 ml 0.1% agarose substrate (20 mM phosphate buffer, pH 7.0), was incubated at 50°C for 40 min. The enzyme activity was determined with the 3,5-dinitrosalicylic acid (DNS) method (Chi et al., 2014) with D-galactose as standard product. One unit (U) is defined as the amount of enzyme to release 1 μg reducing-sugar per minute.

### Characterization of Aga1904

To determine the effect of pH on Aga1904 activity, the enzyme-substrate mixture was incubated in different buffer systems with pH ranging between 4.0 and 11.0 (pH 4.0–7.0, Na_2_HPO_4_-citric acid; pH 7.1–8.9, Tris–HCl; pH 9.0–10.6, glycine-NaOH). To determine the effect of temperature on Aga1904 activity, the enzyme-substrate mixture was incubated at several temperatures ranging from 20 to 80°C. Thermal stability of Aga1904 was determined by measuring residual enzyme activity at optimum temperature and pH after incubating Aga1904 at 40, 50, and 60°C for 0 to 24 h. The effect of metal ions (2 mM) on Aga1904 was evaluated by adding different concentrations of metal ions (Sr^2+^, Ni^2+^, Ca^2+^, Ba^2+^, Mn^2+^, Mg^2+^, Fe^2+^, Fe^3+^, K^+^, Cu^2+^, and Na^+^) to the enzyme-substrate mixture. The activity of Aga1904 without any metal ions was used as the control.

### Kinetic parameter assays

The enzyme activity of Aga1904 with different concentrations of the agarose substrate was measured to obtain the kinetic parameters of Aga1904. The values of the Michaelis–Menten constant (*K*_m_) and maximal reaction rate (*V*_max_) were analyzed using the Lineweaver–Burk double-reciprocal method (Morrison, 2002).

### Analysis of enzymatic hydrolysates of Aga1904

Agarose was incubated with Aga1904 for 0.25, 0.5, 1, 2, 6, 12, and 24 h under standard assay conditions. The reaction was then immediately terminated by putting it in a boiling water bath for 15 min. The reaction solution was centrifuged at 10 000 × *g* for 10 min. The supernatant was concentrated through distillation to approximately 100 ml and centrifuged at 10 000 × *g* for 10 min to remove the insoluble matter, and the supernatant was stored at 4°C for later use.

The enzymolysis products of Aga1904 were analyzed with TLC. Standard samples and the supernatant of the reaction solution were adsorbed to a silica gel plate. The plate was transferred to a chromatography tank containing 15 ml developing agent. After development, the silica plate was removed, air dried and evenly sprayed with color developing agent. After air drying, the silica gel plate was baked in a 90°C oven for approximately 10 min until the color was visible.

### Purification of enzymatic hydrolysates

Agarose was incubated with Aga1904 for 24 h under standard assay conditions. The obtained agaro-oligosaccharides were desalinated, purified and concentrated using a Bio-Gel P-2 column (100 cm × 2 cm). The column was eluted with Millipore water at an elution rate of 12 ml/h and eluting fractions of 2.5 ml were collected automatically. Elution fractions containing sugar were identified using the phenol-sulfuric acid method. These fractions were pooled, lyophilized and used for further analysis and anti-inflammatory assays.

### Evaluation of anti-inflammatory activity of enzymatic hydrolysates

RAW264.7 macrophages were cultured in high-glucose Dulbecco’s Modified Eagle Medium (DMEM), containing 10% (v/v) FBS and 1% (v/v) antibiotics (100 U/ml penicillin and 100 U/ml streptomycin), at 5% CO_2_ and 37°C. When the cell grows to nearly 100%, macrophages were inoculated into a 6/96-well plate at a cell density of about 5 × 10^4^ cells/ml. LPS (1 μg/ml) served as the positive control in all experiments.

We evaluated cytotoxicity using MTT cell viability assays (Zhang et al., 2003). Macrophages were incubated with purified enzymatic hydrolysates (from 50 to 400 μg/ml) for 24 h. The old culture medium was discarded and 100 μL FBS-free medium containing 500 μg/ml MTT solution was added to each well and incubated to form formazan crystals. The medium was then carefully discarded and 50 μL DMSO was added to each well to dissolve the formazan. Absorbance was measured at 490 nm.

Griess reagent is commonly used to determine NO concentration in cell experiment (Giustarini et al., 2008). Incubated RAW264.7 macrophages were inoculated into a 96-well plate and stimulated with 1 μg/ml LPS and the purified enzymatic hydrolysates (between 50 and 400 μg/ml) were added. A drug-free group and an L-NMMA-positive drug group were used as controls. Each group were done in triplicate. The absorbance was measured at 540 nm. The concentration of NO was calculated using a standard curve obtained by plotting the absorbance of various NaNO_2_ concentrations.

To further evaluate the anti-inflammatory activity of agaro-oligosaccharides dominated by neoagarobiose, RAW264.7 macrophages were incubated in 6-well plates with purified enzymatic hydrolysates (between 50 and 400 μg/ml) for 24 h. A drug-free group and an L-NMMA-positive drug group were used as controls. The cell culture medium was centrifuged and commercial ELISA kits were used to determine TNF-α and IL-6 concentrations.

## Results

### Sequence analysis of *aga1904*

**FIGURE 1:**
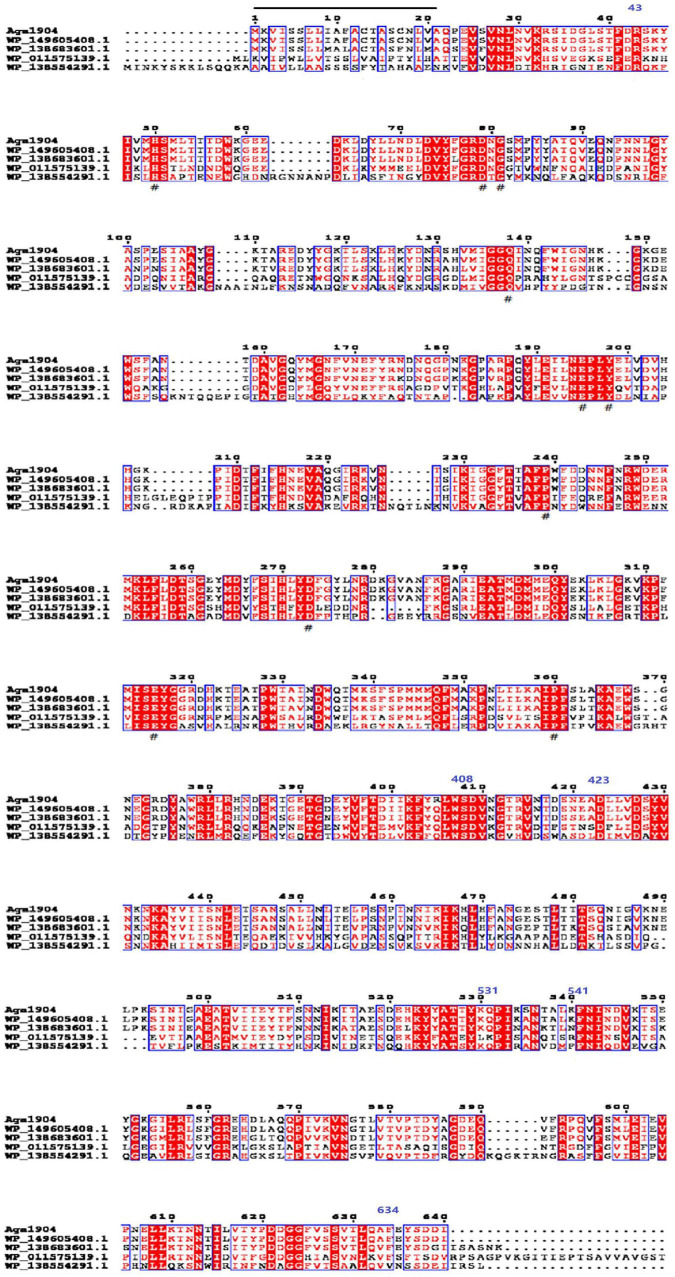
Multiple sequences alignment of Aga1904. “_” is the signal peptide (exists between 1 and 21 amino acids); “#” is the catalytic site; three predict domains: agarase_cat (exists between 43 and 408 amino acids), Porphyrn_cat_1 (exists between 423 and 531 amino acids), and BPA_C (exists between 541 and 634 amino acids).

A novel agarase gene, *aga1904*, was screened from the metagenomic library of macroalgae-associated bacteria of *P. cartilagineum*. The predicted open reading frame (ORF) consisted of 1,923 bp, which encoded a protein of 640 amino acids. The deduced Aga1904 protein had a putative molecular mass of 72 kDa, the isoelectric point is 5.45, and the signal peptide exists between 1 and 21 amino acids. Sequence analysis showed that the encoded protein had three predict domains including, agarase_cat, BPA_C, Porphyrn_cat_1, and belongs to the agarase_cat superfamily (Figure 1). The *aga1904* gene was 1,923 bp long and coded for a 640 amino acid protein with a theoretical molecular weight of 72 kDa. Comparison of the amino acid sequence of Aga1904 and previously reported agarases (WP_138554291.1 and WP_011575139.1) showed 37.1–45.55% sequence identity (Figure 1). Although the percentage identity between Aga1904 and recently submitted sequences WP_138683601.1 and WP_149605408.1 was more than 90%, these sequences are based on metagenomic annotation and characterization studies have not been done yet.

**FIGURE 2:**
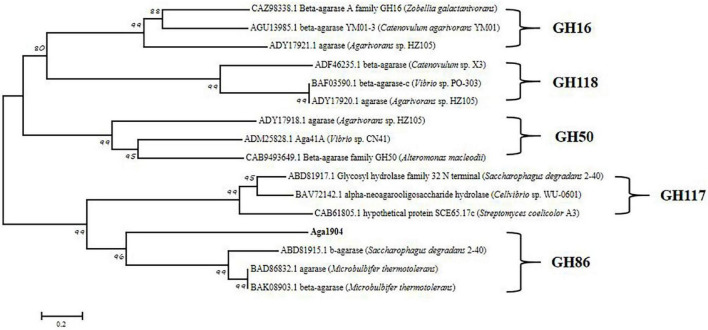
Phylogenetic analysis of Aga1904 (highlighted in bold). The phylogenetic tree was constructed based on the multiple sequence alignment of Aga1904 with all structure-clarified GH16, GH50, GH86, GH117, and GH118 sequences in the CAZy database. The phylogenetic tree generated by the neighbor-joining method. The Genbank accession numbers, protein names and organism names were successively listed in labels.

According to the carbohydrate-active enzyme (CAZy) database, β-agarases are mainly distributed in five different CAZymes families, namely GH16, GH50, GH86, GH117, and GH118 (Ekborg et al., 2006). To further analyze the genetic evolutionary status of Aga1904, a phylogenetic tree was generated with the neighbor-joining method by selecting the amino acid sequences of five reported glycoside hydrolase (GH) families and Aga1904. As shown in Figure 2, Aga1904 and the members of the GH86 family converge into one branch, indicating that Aga1904 belongs to the GH86 family.

### Expression and purification

**FIGURE 3:**
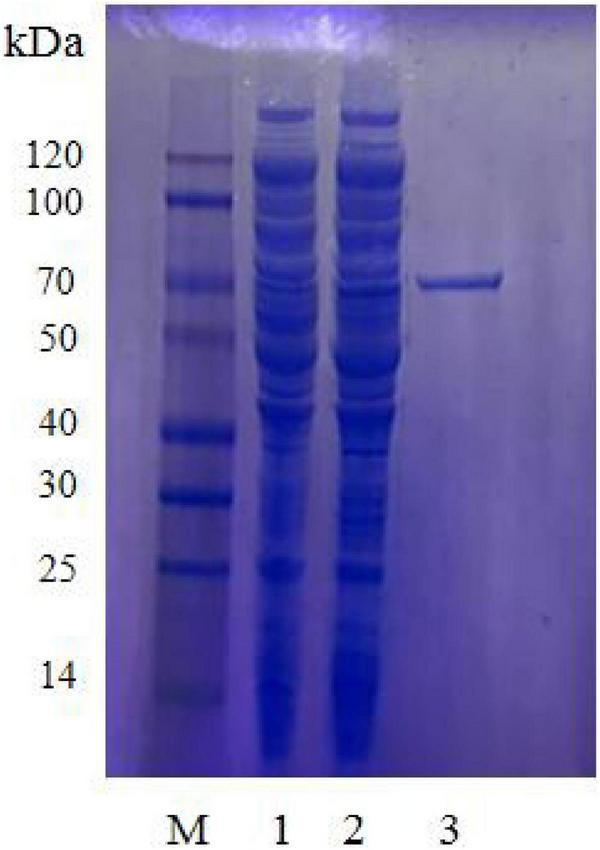
Sodium dodecyl sulfate polyacrylamide gel electrophoresis of Aga1904. M, protein marker; Lane 1, supernatant of recombinant *E. coli* BL21(DE3) without IPTG induction; Lane 2, supernatant of recombinant *E. coli* BL21(DE3) induced by IPTG; Lane 3, purified protein.

We expressed the recombinant plasmid of pET30(a) + *aga1904* in *E. coli* BL21 (DE3) cells by induction with IPTG at a final concentration of 0.5 mM. After purification by Ni-NTA His-tag kit, SDS-PAGE showed a single band, which corresponded to the calculated mass (72 kDa) of Aga1904 (Figure 3). The purified recombinant Aga1904 showed high activity on the agar screening medium (Supplementary Figure 1). The purified recombinant Aga1904 exhibited specific activity levels of 53.22 ± 0.02 U/mg toward the substrate of agarose.

### Characterization of recombinant Aga1904

**FIGURE 4:**
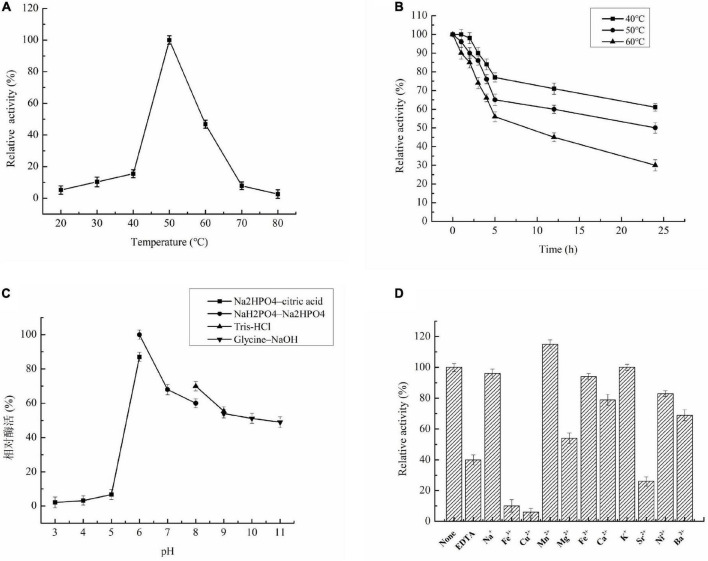
Characterization of recombinant Aga1904. **(A)** Effect of temperatures on enzyme Aga1904 activity; **(B)** the effect of thermostability on Aga1904 at different temperatures and time points; **(C)** effect of pH on enzyme Aga1904 activity; **(D)** effect of metal ions on enzyme Aga1904 activity.

The recombinant Aga1904 retained about 50% of its initial activity between temperatures of 45–60°C, with highest activity seen at 50°C (Figure 4A). Notably, Aga1904 showed high thermostability with almost 55% of its original activity retained after incubation at 40, 50, and 60°C for 5 h. With increasing incubation times, the enzyme activity gradually decreased, but more that 50% of its initial activity was retained after incubation for 24 h at 50°C (Figure 4B).

The optimal pH for Aga1904 was found to be 6.0. Aga1904 has high stability across a broad pH range and retains more than 50% of its maximum activity between pH 7.0 and 11.0 (Figure 4C). Our results revealed that Aga1904 is alkali tolerant, which is an important characteristic for enzymes used for industrial biotransformations.

Recombinant Aga1904 was activated by Mn^2+^, with Mn^2+^ increasing activity by 15%. In contrast, EDTA, Fe^3+^, Cu^2+^, Ba^2+^, Mg^2+^, and Sr^2+^ significantly inhibited activity, with Fe^3+^ and Cu^2+^ dramatically reducing Aga1904 activity (Figure 4D).

### Kinetic parameters of Aga1904

**FIGURE 5:**
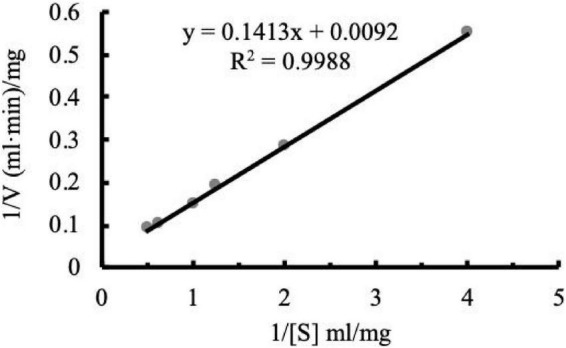
Kinetic parameters of Aga1904.

The Michaelis constant (*K*_m_) is defined as the substrate concentration at which the reaction rate reaches half of the maximum reaction rate. *K*_m_ values are both enzyme and substrate specific. Furthermore, *K*_m_ values reflect enzyme-substrate affinity, with smaller *K*_m_ values indicating greater affinity. Figure 5 shows the Lineweaver–Burk double-reciprocal plot of Aga1904. The *V*_max_ of Aga1904 was 108.70 mg/ml min and the *K*_m_ was 15.36 mg/ml.

### Analysis of enzymatic hydrolysates of Aga1904

**FIGURE 6:**
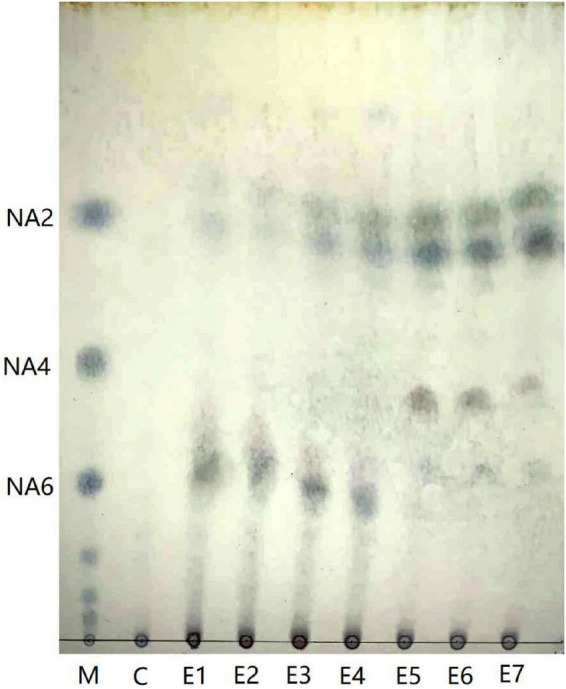
Thin-layer chromatogram of hydrolysis products produced by purified Aga1904. Lane M: NA2, NA4, and NA6, standard samples of neoagarobiose, neoagarotetraose, and neoagarohexaose, respectively; Lane C: control, agarose without Aga1904 for 1 h in standard assay conditions; Lane E1–E7: agarose incubated with purified Aga1904 for 0.25, 0.5, 1, 2, 6, 12, and 24 h in standard assay conditions.

**FIGURE 7:**
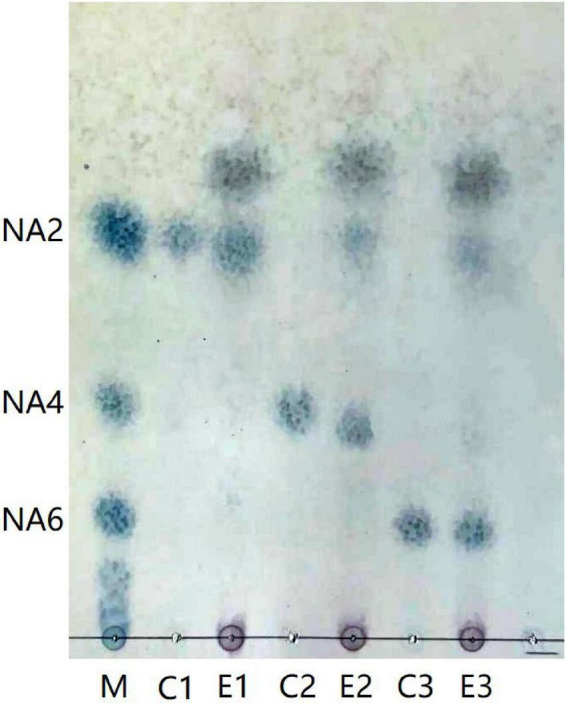
Thin-layer chromatogram of purified Aga1904 hydrolyzed neoagarobiose, neoagarotetraose, and neoagarohexaose standard samples. Lane M1: NA2, NA4, and NA6, standard samples of neoagarobiose, neoagarotetraose, and neoagarohexaose, respectively; Lane C1: control, neoagarobiose without Aga1904 for 6 h in standard assay conditions; Lane E1: neoagarobiose incubated with purified Aga1904 for 6 h in standard assay conditions; Lane C2: control, neoagarotetraose without Aga1904 for 6 h in standard assay conditions; Lane E2: neoagarotetraose incubated with purified Aga1904 for 6 h in standard assay conditions; Lane C3: control, neoagarohexaose without Aga1904 for 6 h in standard assay conditions; Lane E3: neoagarohexaose incubated with purified Aga1904 for 6 h in standard assay conditions.

Aga1904 degrades agarose to neoagarobiose, neoagarotetraose, and neoagarohexaose (Figure 6). The degradation product at 0.25 to 2 h was mainly neoagarohexaose and its production gradually decreased over time. At 6 h, neoagarobiose and neoagarotetraose began to appear. Over time, the neoagarobiose gradually increased with the reduction of neoagarotetraose. These results indicate that Aga1904 is an exo-agarase. To further verify the enzymatic hydrolysis type, Aga1904 was incubated with neoagarobiose, neoagarotetraose, and neoagarohexaose separately for 6 h (Figure 7). Aga1904 was found to degrade both neoagarotetraose and neoagarohexaose into neoagarobiose. Surprisingly, Aga1904 could further degrade neoagarobiose into monosaccharides.

### Evaluation of anti-inflammatory activity of enzymatic hydrolysates

**FIGURE 8:**
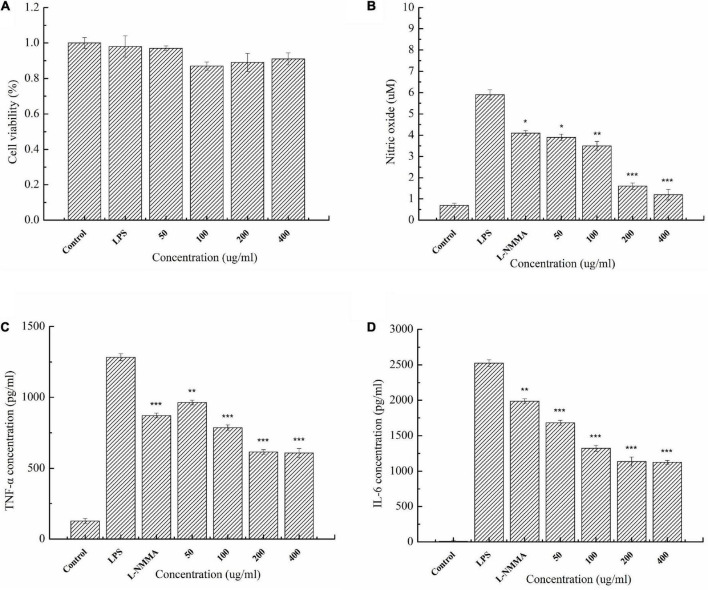
Cytotoxicity of enzymatic hydrolysates on RAW264.7 macrophages **(A)**. Effect of agaro-oligosaccharides on NO production **(B)**. Effects of agaro-oligosaccharides on extracellular secretion of TNF-α **(C)**, and IL-6 **(D)**. Data are presented as the mean ± SE (*n* = 3) from independent experiments. Significance: *P < 0.05, **P < 0.01, and ***P < 0.001.

The cytotoxicity of agaro-oligosaccharides produced by Aga1904 was evaluated by treating RAW264.7 macrophages with various concentrations of the enzymatic hydrolysates. Concentrations at which RAW264.7 macrophage cell viability was above 70% were generally considered to be non-toxic. Even at concentrations of 400 μg/ml, no significant effect on LPS-induced RAW264.7 cell viability was observed (Figure 8A). Therefore, agaro-oligosaccharide concentrations of 50–400 μg/ml were used for the anti-inflammatory assays.

The anti-inflammatory activity of the enzymatic hydrolysates was investigated. It was found that agaro-oligosaccharides could significantly attenuate the secretion of inflammatory mediators, such as NO, and cytokines, such as TNF-α and IL-6, in LPS-activated macrophage RAW264.7 cells (Figures 8B–D). Moreover, the experimental results showed that agaro-oligosaccharides had a greater inhibitory effect than the L-NMMA control group.

## Discussion

At present, the large-scale preparation of high-purity oligosaccharides still presents a technical challenge (Yun et al., 2016). The conditions for the large-scale production of oligosaccharides by chemical and physical methods are difficult to control and easily pollute the environment, and the prepared products have low purity. In contrast, enzymatic hydrolysis has the advantage of strong substrate specificity, high product specificity, and mild reaction conditions, ensuring the stability of oligosaccharide products and enabling the preparation of high-purity oligosaccharides in large quantities (Yang et al., 2014). Preparation of oligosaccharides by polysaccharide-degrading enzymes therefore have promising prospects for broad applications. Recently, many agarases have been obtained from microorganisms from different environments, including sediment, seawater, and macroalgae. In the past 20 years, multiple studies have focused on macroalgae-associated bacteria and their production of polysaccharide-degrading enzymes (Tropeano et al., 2012, 2013). However, there are few reports about polysaccharide-degrading enzymes obtained from the macroalgae-associated bacteria collected from Antarctica. Furthermore, active screening of culturable microorganisms is still the primary method used to obtain polysaccharide-degrading enzymes (Tringe and Rubin, 2005; Tian et al., 2012; Bhattacharyya et al., 2014). However, the recent increase in available metagenomic data has provided a new way of screening for novel polysaccharide-degrading enzymes produced by unculturable microorganisms. In this study, a novel β-agarase gene a*ga1904*, belonging to the GH86 family, was screened from the metagenomic library of Antarctic macroalgae-associated bacteria and was expressed in *E. coli* BL21 (DE3).

In this study, Aga1904, a novel GH86-family agarase, was well characterized. Aga1904 showed high thermostability and alkali tolerance. These are important characteristics for enzymes utilized in biotransformations and industrial production because polysaccharide substrates are colloidal and viscous at low temperatures and high concentrations, which impedes the enzyme-substrate binding efficiency (Li et al., 2018). The N-terminal sequence is usually closely related to thermal stability of polysaccharide degrading enzymes. Many researchers have modified the N-terminal of cellulase and xylanase through biochemical methods to improve their thermal stability, including N-terminal substitution (Yin et al., 2013), amino acid site mutation (Zhang et al., 2014), etc. Moreover, the disulfide bonds structure is another major means to stabilize protein conformation to improve the thermal stability of enzymes. Therefore, we will reveal and improve the thermostability of Aga1904 by the way of amino acid site mutation or introducing disulfide bonds into protein structure in the following work. The effects of the metal ions on Aga1904 activity were variable. Fe^3+^ and Cu^2+^ dramatically reduced Aga1904 activity, which corresponds to previous studies that reported the inhibitory effect of Cu^2+^ on the activity of Amy19 (Li et al., 2018) in the GH70 family and AgaXa (Xie et al., 2013) in the GH118 family. Cu^2+^ decreases enzyme activity by binding with the thiol group in the active site of enzymes (Murashima et al., 2002). Similar with previous study, sequence analysis showed that there are two thiol-containing amino acids (Cys) existed at the sequence of Aga1904, accounting for 0.3% of the amino acid composition.

The *K*_m_ value of Aga1904 (15.36 mg/ml) was similar to the *K*_m_ value of the agarases from *Agarivorans albus* OAY02 (15.38 mg/ml) (Yang et al., 2014), and lower than the *K*_m_ values of the AgaD50 (41.9 mg/ml) (Dong et al., 2016) and rEAgaO (1437 mg/ml) (Dong et al., 2016). Our results showed that Aga1904 has a good substrate-binding ability and its good kinetic parameters are also advantageous for industrial application.

There are few reports on the biological activity of neutral agaro-oligosaccharides prepared by enzymatic hydrolysis. We prepared agaro-oligosaccharides by enzymatic hydrolysis of agarose by Aga1904. The resultant degradation products were mainly neoagarobiose, neoagarotetraose, and neoagarohexaose. Moreover, our results showed that after 24 h the enzymatic hydrolysates are dominated by neoagarobiose, which can greatly simplify the preparation, separation, and purification process of agaro-oligosaccharides. The purified enzymatic hydrolysates were further investigated for anti-inflammatory activity.

Cytokines are important indicators of the inflammatory response *in vivo*, being released in response to infection, inflammation, and trauma (Dinarello, 2000). However, over-production of pro-inflammatory cytokines, such as TNF-α and IL-6, can accelerate the occurrence of inflammatory diseases (Siebert et al., 2015). Excessive production of pro-inflammatory cytokines can also cause a strong inflammatory response, leading to shock and, in serious cases, death (Dinarello, 2000). Inhibition of pro-inflammatory cytokine secretion is an important treatment method of various inflammatory diseases (Papadakis and Targan, 2000). In recent years, there has been increasing research into anti-inflammatory substances from natural sources, and many oligosaccharides have excellent anti-inflammatory activities. Algino-oligosaccharides can inhibit the binding of LPS to Toll-like receptor 4 (TLR4) of RAW264.7 cells, thereby inhibiting the excessive activation of NF-κB and MAPK signaling pathways, and thus effectively inhibiting inflammation and neuroinflammation (Zhou et al., 2015a,b). Moreover, the anti-inflammatory activities of L-AHG, D-Gal and neoagarobiose (NA2) have been tested in LPS-stimulated RAW264.7 cells, and only L-AHG at a concentration over 100 μg/ml showed suppressive effect on nitrite production (Yun et al., 2013). In this study, LPS-induced RAW264.7 cells were used to construct an *in vitro* inflammatory model. LPS binds to TLR4, activating NF-κB and MAPK signaling pathways, which leads to the production of excessive inflammatory mediators, resulting in a strong inflammatory response (Li et al., 2014). We found that agaro-oligosaccharides dominated by neoagarobiose could effectively inhibit excessive NO, as well as TNF-α and IL-6, production in LPS-induced RAW264.7 cells. The enzymatic hydrolysates (dominated by neoagarobiose) showed significantly higher inhibitory activity against LPS-induced RAW264.7 cells than L-NMMA. L-NMMA is a congener of L-arginine, competes with L-arginine for the binding site of nitric oxide synthase (NOS), and thereby inhibits all three kinds of NOS. The results suggest that enzymatic hydrolysates of Aga1904 have potential application prospects as natural anti-inflammatory agents.

To our knowledge, this is the first report of an enzyme screened from the metagenomic library of macroalgae-associated bacteria that can effectively degrade agarose to obtain agaro-oligosaccharides with excellent anti-inflammatory activity. Our study provides a theoretical basis for developing agaro-oligosaccharides as a potential health food or drug to treat inflammation.

## Data availability statement

The amino acid sequence of β-agarase Aga1904 has been deposited in GenBank under the accession number ON723902.

## Author contributions

JL collected the samples, designed the research, supervised the project, and analyzed the data. XG conducted experimental work and drafted the manuscript. LZ, JT, QZ, and LF assisted with experiments. All authors contributed to the article and approved the submitted version.

## Footnotes

1
http://blast.ncbi.nlm.nih.gov/Blast.cgi

2
http://www.lynnon.com/
