# Supplementary material for: Characterization of a novel β-agarase from Antarctic macroalgae-associated bacteria metagenomic library and anti-inflammatory activity of the enzymatic hydrolysates
Source: Front Microbiol. 2022 Sep 2;13:972272. doi: 10.3389/fmicb.2022.972272 (PMC9478344; doi:10.3389/fmicb.2022.972272)
Supplement: Supplementary file 1 [file Data_Sheet_1.doc]

Supplementary Material

**Supplementary Figure 1.** Positive clones of purified recombinant Aga1904.


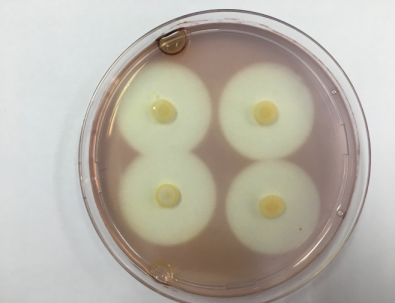


Fig. S1
